# REATIVA: An Efficient Health Promotion Program during Retirement Transition

**DOI:** 10.3390/ejihpe12090095

**Published:** 2022-09-11

**Authors:** Helena Loureiro, Margarida Silva, Ana Paula Camarneiro, Ana Teresa Pedreiro, Aida Mendes

**Affiliations:** 1 Health School, University of Aveiro, 3810-193 Aveiro, Portugal; 2 Nursing School of Coimbra, Avenida Bissaya Barreto, 3046-851 Coimbra, Portugal; 3 Health Sciences Research Unit: Nursing, Rua Dr. José Alberto Reis, 3000-232 Coimbra, Portugal

**Keywords:** health promotion program, retirement, active aging

## Abstract

Retirement is a major life transition in adulthood that can cause vulnerability in individuals and their families. REATIVA is a face-to-face health intervention program that aims to promote the perception of self-efficacy and facilitate the transition to retirement of individuals and families. This article presents the efficiency of this program. A quasi-experimental study was conducted on 56 new retirees and families enrolled in the Portuguese National Health Service. The efficiency of the program was evaluated with the General Self-Efficacy (GSE) scale, which assesses self-efficacy, and the Retirement Adaptation Perception (EPFAR) scale, which assesses the perception of adaptation to retirement. The data were processed in IBM SPSS 27 software. An average positive change was found regarding the GSE and EPFAR in all participants of the REATIVA program. Notably, the MANOVA test with Greenhouse–Geisser correction revealed a significant effect of the program over time in the EPFAR scale (F = 17.405, *p* = 0.001; η2 = 0.554; PO = 0.982). The REATIVA program was found to be efficient in the promotion of individual and family health during the transition to retirement as an active and healthy aging process. New methodologies and intervention strategies were identified that could improve the efficacy of the program; namely, the involvement of more family members and using a blended approach.

## 1. Introduction

The transition to retirement is an event in the human lifecycle that occurs in late midlife and is characterized by a process of adaptation to change in a temporal space, determined by how its protagonists achieve systemic homeostasis [1]. It involves losses and gains that trigger an ambivalent perception of positive and negative feelings and is accompanied by a differentiated degree of stress according to the cause or motivation behind its occurrence [2]. Thus, if the transition to retirement was desired and/or previously planned, the perception of stress may be lower than when it occurs unexpectedly and/or outside the individual’s will (e.g., compulsory or early retirement). In this same context, it is also important to mention that the degree of satisfaction the person had with their job and/or the “arrival of retirement” may influence how they adjust to this new experience, those who least look forward to the proximity of retirement generally being those most adapted and satisfied with their jobs [3].

However, regardless of the adaptation phase and/or the perception they present in this period of their lives, both individuals and families who experience the transition to retirement seem to agree that it is a life-changing event that requires greater adaptive effort in how to act, be, and feel as a family [4]. This transition has repercussions in the most diverse areas of human development and family dynamics and exposes these systems to a high degree of vulnerability for health [5]. Thus, plans should be made to minimize the constraints that may arise from it.

Perceived general self-efficacy refers to people’s perceptions of their competence in performing a certain task to achieve a certain goal or outcome, which influences how they feel, think, and act, these being the foundation of human decision-making and perseverance [6]. Taylor and Shore [7] stated that high levels of self-efficacy are associated with the ability to adjust successfully to retirement, as it is a predictor of retirement planning. In contrast, individuals with low self-efficacy are less able to exploit the opportunities provided by the system and, consequently, struggle more to adapt to transitions [6]. Self-efficacy influences the perception of self and environment and, in turn, affects the dynamics of adjustment to retirement [8]. A study by Dyck, Cardon, and De Bourdeaudhuij [9] demonstrated that, at the beginning of retirement, high levels of self-efficacy are associated with an increase in physical activity. Therefore, health interventions should focus on self-efficacy because the avoiding a sedentary lifestyle is fundamental for maintaining active aging.

However, because this perception may vary due to instability and influence, individuals with high self-efficacy tend to be proactive in structures. In contrast, when self-efficacy is low, individuals are less able to exploit the opportunities provided by the system [10].

These considerations indicate the relevance of analyzing the results of other variables that constitute resources for successful retirement,^,^ such as awareness of the new retiree status, knowledge about ageing adaptation process, family support and community network support [3]. Therefore, programs that promote a successful transition for events that occur in middle age can help facilitate active and healthy aging.

This article aims to present the results of the assessment of the efficiency of the REATIVA program^1^ in promoting active and healthy aging in individuals and couples experiencing the transition to retirement.

## 2. Materials and Methods

### 2.1. Research Design

**Figure 1:**
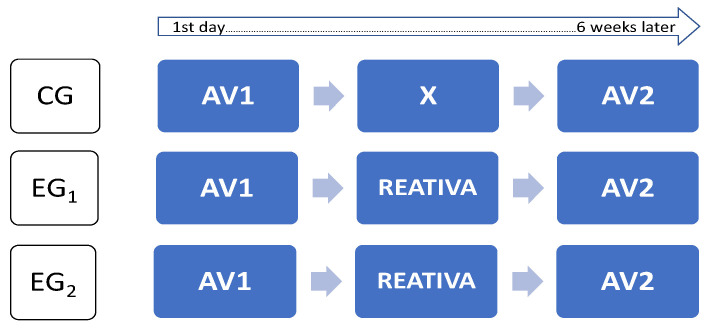
Research design for assessing the efficiency of the REATIVA program.

A quasi-experimental intervention-action study was conducted with three distinct groups: one control group (CG) without any prescriptive intervention who filled in the assessment questionnaires in the pre-test and post-test assessments; and two experimental groups (EG_1_ and EG_2_), who were both submitted to the REATIVA program and filled in the assessment questionnaires in the pre-test (Av1) and post-test (Av2) assessments (Figure 1).

Different participants were allocated to the two experimental groups: EG_1_ consisted of new retirees who participated individually and EG_2_ included couples with at least one newly retired spouse.

The inclusion criteria for selecting the groups were:

- For the EG_1_ and CG, “to be retired for less than five years” and “to participate individually”;
- For the EG_2_, “to participate as a couple”, with at least one of the spouses retired for less than five years, and “to live as a couple” (married or in a *de facto* union) before the transition to retirement of one of the spouses.

After the intervention’s objectives were explained and the individuals meeting the inclusion criteria were identified, they were contacted by phone and invited to participate in the REATIVA program.

### 2.2. REATIVA Program and Implementation

Before implementing the REATIVA program:

- Formal interviews were carried out with the coordinating teams of the health units where the program was to be implemented to present the objectives and the intervention model and to request their collaboration in identifying potential participants and their authorization to use their facilities for this study;
- All participants signed the informed consent form after having been informed of all the procedures and ethical principles inherent to the participation in a research study of this nature.

The REATIVA program [1] consisted of 12 face-to-face sessions, 90 min each, twice a week, addressing the topics of Retiree Status (two session), Mental Health (two sessions), Support Networks (one session), Health and Aging (two sessions), Economic Management (one session), Family and Conjugality (two sessions), and Family and Parenting (two sessions). Each session had a plan that included an introduction, a middle part, and a conclusion and used active methodologies [1]. Each session was conducted by an expert in each area who belonged to the research team.

For the purpose of conducting an immediate assessment of the efficiency of this program, the assessment instruments were applied before and after the program’s implementation to measure the GSE and EPFAR variables.

### 2.3. Participants

Participants were recruited through five randomized health-care centers in Portugal. The sample was selected in a first phase through Family Nurse identification. After the initial contact of 75 participants, 57 accepted the offer to participate in the study. After the beginning of the sessions, one participant decided to drop out of the program for typical reasons. A total of 56 individuals participated in the study: 15 in the EG_1_ (26.8%), 12 in the EG_2_ (21.4%), and 29 in the CG (51.78%). Considering this was a quasi-experimental study, the participants were randomized based on the homogeneity present for the sociodemographic variables of age, gender, and education level.

Regarding the overall sociodemographic characterization, the participants of the three groups were aged between 48 and 72 years (X= 62.8 years; SD = 3.1 years), mostly female (57.1%), married or in a *de facto* union (85.7%), and had more than 9th grade but no higher education (39.3%). Considering only the retirees (87.5%) and the length of retirement, 14.2% had been retired for 3–4 years, 8.9% for less than 1 year, 8.9% for 4–5 years, 10% for 1–3 years, and 3.6% for more than 5 years. During their working lives, 32.1% of the participants had administrative or similar jobs; 10.7% were specialists in intellectual and scientific professions; 10.7% were unskilled workers; 7.1% were technicians and intermediate level workers; 5.4% were manual laborers, artisans, or similar; 3.6% were senior public administration employees, managers, and company executives; and 3.6% were farmers and skilled workers in the agricultural and fishing sectors. When questioned about their job satisfaction, 23.2% were very satisfied, 16.1% were satisfied, 8.9% were dissatisfied, and 1.8% were very dissatisfied. When asked how satisfied they were at the time with the prospect of retiring, 16.1% reported being satisfied, 12.5% reported being very satisfied, and 8.9% reported being dissatisfied.

### 2.4. Measures

#### 2.4.1. General Self-Efficacy Scale

Schwarzer and Jerusalem’s (1995) General Self-Efficacy Scale (GSE) [11], based on Albert Bandura’s construct, measures perceived self-efficacy in coping with new and different challenges or adverse events in the different domains of human functioning. Multiple studies over two decades have found that this one-dimensional scale can be used as a predictor of adaptation to life changes and as an indicator of motivational performance at any time. This study used the Portuguese version of the GSE, validated by Nunes, Schwarzer, and Jerusalem (1999) [12], an instrument with 10 closed-ended items rated on a four-option Likert-type scale, in which 1 corresponds to “Not at all true”, 2 to “Hardly true”, 3 to “Moderately true,”, and 4 to “Exactly true”.

#### 2.4.2. Positioning for Adaptation to Retirement

The REATIVA project (PTDC/MHC-PSC/4846/2012) research team developed the Positioning Scale for Adaptation to Retirement (EPFAR) based on the evidence gathered during the previous stages of its development. Focus groups were conducted with new retirees [13] and interviews with couples [14] to identify the main changes/difficulties of adaptation to retirement experienced by new retirees. The EPFAR was also based on the comprehensive reading of books, articles, academic studies, and expert opinion papers on adaptation to retirement and aging, which resulted in a systematic literature review [15].

This instrument consisted of 28 items distributed across the seven dimensions corresponding to the main factors that cause greater vulnerability during retirement transition. Four items were allocated to each of the topics addressed in the intervention (Retiree Status, Mental Health, Support Networks, Health and Aging, Economic Management, Family and Conjugality, and Family and Parenting). This scale has shown good reliability when validated with the Portuguese population [16].

### 2.5. Data Analysis

Before the statistical analysis, the reliability of the instruments for assessing the evolution of the GSE and EPFAR variables was determined by measuring the internal consistency of the items that the participants responded to at the pre-test assessment (Av1) and post-test assessment (Av2). The GSE scale showed overall good psychometric characteristics, with total Cronbach’s alpha values of 0.884 (Av1) and 0.742 (Av2). The EPFAR showed high reliability, with total Cronbach’s alpha values of 0.865 (A1) and 0.810 (A2).

Then, the distribution of these variables was determined through the test of adherence to normal distribution, which revealed that both the GSE (total GSE: Av1(K-S = 0.08; *p* = 0.784); Av2(K-S = 0.11; *p* = 0.450)) and the EPFAR variables (total EPFAR: Av1(K-S = 0.11; *p* = 0.488); Av2(K-S = 0.12; *p* = 0.327)) had normal distributions in both assessments.

Descriptive statistical analysis was carried out with measures of central tendency (mean (M) and mode (Mo)), location (minimum (Min.) and maximum (Max.)), and dispersion (range (R) and standard deviation (SD)) used to determine how the GSE and EPFAR variables evolved, as well as a paired-samples *t*-test. The significance level was set at *p* = 0.05.

## 3. Results

### 3.1. General Self-Efficacy (GSE) Scale

Student’s *t*-test for paired samples was used to compare the self-efficacy means before and after implementing the program and showed a positive and statistically significant evolution of the GSE variable in the experimental groups (Table 1), which demonstrates the program’s benefits for the participants.

The results were favorable in both the EG_1_ (*t* = 3.287; *p* = 0.005) and the EG_2_ (*t* = 2.469; *p* = 0.033). A slight decrease in the GSE mean was also observed in the CG, but the differences in the GSE values were not significant between the two assessment moments (*t* = −0.668; *p* = 0.510). The significant change in the self-efficacy of retirees submitted to the REATIVA program suggests that they began to believe more in their personal abilities.

An analysis per item found that items 4 (“I am confident that I could deal efficiently with unexpected events”) and 8 (“When I am confronted with a problem‚ I can usually find several solutions”) were responsible for the level of significance obtained in EG_1_.

In EG_2_, the significant increase in self-efficacy was caused by item 7 (“I can remain calm when facing difficulties because I can rely on my coping abilities”) and item 9 (“If I am in trouble‚ I can usually think of a solution”). Here, the items regarding more passive coping strategies—namely, calmness and thoughtfulness—were further strengthened after the program was implemented.

### 3.2. Positioning Scale for Adaptation to Retirement

The EPFAR was assessed in the EG_1_ and EG_2_ before (Av1) and after (Av2) the implementation of the REATIVA program (Table 2). A similar assessment was carried out in the CG, but the new retirees of this groups were not submitted to any intervention.

All groups showed a positive mean evolution in this variable (EG_1_:M(Av2-Av1) = 0.367; SD = 0.366/EG_2_:M(Av2-vA1) = 0.237; SD = 0.469/CG:M(Av2-A1) = 0.093; SD = 0.325). However, as this evolution was only significant in EG_1_ (*t* = 3.906; *p* = 0.002), it seems that the REATIVA program effectively improved the perceived adaptation to retirement of the individual participants.

An analysis of each group and each dimension of the scale revealed that the program did not have the same effect on all the participants. Moreover, the characteristics of the group to which the participants were allocated seemed to have influenced how their perceptions evolved.

Thus, in the “Retiree Status” dimension, the results obtained for the item “I can identify goals for my future life” (EG_1_: M(Av2-Av1) = 0.66; *t* = 2.320; *p* = 0.036/EG_2_: M(Av2-Av1) = 0.51; *t* = 2.283; *p* = 0.046) suggested that the program was effective in achieving one of its main objectives: facilitating the perception of efficacy in dealing with the changes and challenges inherent to the retiree status of the participants.

In the “Mental Health” dimension, the results obtained for the item “I feel in harmony with my current life” (EG_1_: M(Av2-Av1) = 0.60; *t* = 3.154; *p* = 0.007/EG_2_: M(Av2-Av1) = 0.15; *t* = 3.614; *p* = 0.005) highlighted the program’s psycho-emotional effect on its participants. In addition, the program also contributed to an increase in the participating couples’ efficacy in coping with the stress caused by this experience (“I can manage everyday stress” (EG_2_: M(Av2-Av1) = 0.727; *t* = 3.730; *p* = 0.004)).

The results in the “Support Networks” dimension showed that the program was effective in providing the individual participants with a broader understanding of the community resources available to them for their adaptation needs (“The resources in my community are sufficient” (EG_1_: M(Av2-Av1) = 0.67; *t* = 2.646; *p* = 0.019)). Nonetheless, EG_2_ participants did not have the same perception (M(Av2-Av1) = 0.45; *t* = 0.690; *p* = 0.506), which raises questions about the diversity and characteristics of the current supply of resources aimed at couples.

Regarding the “Economic Management” dimension, the program’s empowerment effect on individual participants was also evident, reflected by their perception of an increase in expense planning (“I make a monthly plan of my expenses” (EG_1_: M(Av2-Av1) = 0.47; *t* = 2.168; *p* = 0.048)).

The participants in the REATIVA program also revealed an evolution in all items of the “Health and Aging” dimension.

In the “Family and Conjugality” dimension, the positive evolution observed in the item “I am sexually satisfied” (EG_1_: M(Av2-Av1) = 0.60; *t* = 2.553; *p* = 0.023 / EG_2_: M(Av2-Av1) = 0.02; *t* = 2.764; *p* = 0.020) shows that the program promoted a greater demand for and sharing of intimacy in its participants. The program also seems to have contributed to the perception of more effective communication among couples (“In my relationship, there is dialogue and sharing” EG_2_: M(Av2-Av1) = 0.04; *t* = 2.390; *p* = 0.038) and of conjugality in life projects among the new retirees who participated individually (“When I decide with my partner, I achieve more and better” EG_1_: M(Av2-Av1) = 0.73; *t* = 2.955; *p* = 0.010).

In the “Family and Parenting” dimension, the perception of support and assistance focused on grandchildren became evident in both groups after the program’s implementation (EG_1_: M(Av2-Av1) = 0.60; *t* = 2.806; *p* = 0.014/EG_2_: M(Av2-Av1) = 0.69; *t* = 2.319; *p* = 0.043). This supporting attitude was expressed in the greater perception of happiness that grandchildren brought to their grandparents (“My grandchildren make me happy” EG_1_: M(Av2-Av1) = 0.73; *t* = 3.214; *p* = 0.006)).

## 4. Discussion

The results demonstrate that the REATIVA program proved to be efficient in achieving its purpose; namely, in empowering new retirees to have a better perception of their self-efficacy in and adaptation to the transition to retirement.

Although there was a positive evolution in perceived self-efficacy in all groups, the results revealed a higher and statistically significant evolution in the program’s participants. They began to believe more in their abilities to successfully initiate and carry out specific tasks that may require effort and perseverance against adversities, according to the initial definition by Bandura [6].

Taylor-Carter and Cook [17] stated that self-efficacy predisposes individuals to engage in proactive strategies that allow them to master the role changes inherent to the transition to retirement. Thus, if individuals increased self-efficacy after participating in a retirement-promotion program, those who adopted the program should have had *a priori* higher levels of self-efficacy than those who did not want to participate in it.

According to Bandura [18], self-efficacy beliefs are protective as they prevent individuals from confronting threatening situations and/or situations for which they do not have the necessary skills and control. Moreover, self-efficacy influences individuals’ relationship with the social system in which they are integrated by determining how they interpret and act within that system. The same author [18] states that individuals with high perceived self-efficacy are proactive in their attitudes and actions, which does not happen with individuals with low perceived self-efficacy, who are less able to explore the system opportunities. These considerations point to the relevance of analyzing results from other variables [3,9] that constitute resources for a successful retirement in order to examine the efficiency of the REATIVA program.

The significant increase in self-confidence and development of active problem-solving strategies contributed the most to developing perceived self-efficacy. This suggests that self-efficacy should be considered a facilitating factor for working individuals to feel more comfortable in their decision to retire and improve post-retirement adjustment [8,19].

Similarly to the evolution observed in the couples who participated in the program, the retirees perceived a significant increase in the use of coping strategies, such as reflection and calmness, to face the adaptive changes brought about by the transition experience. It may have been the result of the empowerment in perceived self-efficacy by the REATIVA program. Conjugality plays an essential role because the presence of the spouse may have contributed to a drive to achieve goals and, consequently, improve these skills. Negrini et al. [20] stated that personal feelings of competence primarily influence adults’ reactions to life transitions and impact their preferences in and decisions about retirement.

Moreover, if the results obtained by assessing the evolution of perceived self-efficacy suggest the empowering effect of the REATIVA program, the same positive evolution observed in the EG regarding the positioning for adaptation to retirement reinforces its effectiveness as a facilitator of the transition to retirement. Several authors [14,15] have suggested that primary health care is a national health service structure where professionals have more knowledge about the dynamics of individuals, families, and communities, so it is their responsibility to implement health-promotion programs that facilitate life transitions.

These findings demonstrate that self-efficacy is an essential psychological variable in adaptive processes. For example, Negrinia et al. [20] proved that self-efficacy is associated with low pre-retirement anxiety, and Van Dyck et al. [9] linked high self-efficacy to more exercise practices and less sedentary lifestyles after retirement. Therefore, it is important to assess self-efficacy early to know the mental health status of new retirees, aiming for improved well-being and overall better health.

The evolution observed in the participants of the REATIVA program also suggests that this intervention boosted these individuals’ acceptance of their retiree status, which eases the adaptation to the impact of these changes [3].

A similar effect was observed in the mental health dimension, with evidence showing the importance of monitoring the psycho-emotional health of the individuals involved in this transition to promote their resilience and well-being [2]. Further, the fact that this result was more significant in new retirees who participated in the REATIVA program as a couple confirms the importance of conjugal relationships in this transition [15].

Considering the support network dimension, the knowledge acquired about the resources available in their community seems to have contributed to their social and health empowerment. In the same context, and because the participating couples did not have the same perception, it is worth noting that resources targeting the marital subsystem, particularly the older couples, are still incipient in modern societies.

The REATIVA program was equally relevant in promoting economic literacy through the perception of a change in attitude about planning expenses. Although some participants during the prototype’s implementation [21] mentioned that this topic should have been presented at an earlier age because they currently considered themselves capable of managing their finances, reflecting on their daily spending practices and reviving the balance sheet and budget planning exercises provided by the program proved efficient and beneficial.

Although there was a positive evolution in all the items of the health and aging dimension, the statistical analysis revealed that this perception might not be directly attributable to the program’s implementation. It is difficult to challenge certain beliefs and myths related to health behaviors that have crystallized over a lifetime and even more so to discuss the issue of aging and associated morbidity or dependence. It is an unappealing theme in this transition, mainly because, as observed during the program’s implementation, the participants undergoing this transition do not see themselves as elderly (which indeed they are not). Nevertheless, it is crucial to understand that this process is progressive, affects all individuals, and, like other life processes, implies becoming aware of it and acting to promote balance [2,14].

The REATIVA program seems equally relevant in raising awareness and developing skills that promote conjugality. This study’s results confirm the need for middle-aged couples to reflect and work on their conjugality, which often falls into the background due to work, parenting, and other life demands [21].

Finally, the program also raised new thoughts about the development of parenting, particularly giving meaning to intergenerationality. In a study conducted with 432 new retirees, Loureiro^4^ found that, after the spouse, these individuals perceived grandchildren as a relevant source of support during their transition. Furthermore, due to the emotional nature of this relationship, it was also confirmed that reinforced relationships between grandparents and grandchildren are a major source of pleasure and affection during a stage of life that retirement makes vaster. However, because the closeness between the two generations often depends on the children’s request, these programs are also relevant for promoting the grandparents’ integration into the family life and, consequently, into successful transgenerationality [3]. Besides the happy relationship with grandchildren, retirees with descendants expect to share leisure activities with their children. They also help their children regardless of the effort involved because they need it. In turn, retirees expect their children to help them in the future by being present, providing affection, and caring for them [22].

One of the aspects that could be used to promote intergenerationality are new technologies and the development of competencies in the use of these technologies, since new generations are enthusiastic about it and can be facilitators in teaching their grandparents. This could be a methodological strategy implemented by REATIVA.

Some studies have proven that digital health may help older adults improve several health determinants [23]. Although REATIVA was a face-to-face program, in the future, it could have an online component, aimed at supporting healthy and active living for individuals who are transitioning to retirement, as recommended by other studies [24]. This would also make it possible for more individuals access it at the same time that they are developing and improving new skills.

### Study Limitations and Recommendations for Future Research

This study encountered several limitations, such as the sample size. Another was the cultural bias in the variables studied, as they were context-specific, multifactorial, and likely to vary quite significantly depending on the group’s embedded cultural factors. For example, in Portugal, family support is very relevant for individual development, which may not be the case for individuals from other countries. Thus, result interpretation should consider the lack of generalizability, and cultural adaptations are needed if the program is implemented in other countries. In addition, the instruments used only provide quantitative data. Regarding the EPFAR scale, it is likely distorted by social desirability bias, making it difficult to explore an increasingly deep-rooted issue.

We present some recommendations for future research related to the above-mentioned limitations. Future similar studies should use a larger sample and compare variables in populations from different locations to avoid sample size limitations and cultural bias. In addition, adequate instruments should be used to analyze qualitative aspects for better result interpretation. It would also be interesting to study the follow-up of the retirees that have participated in the REATIVA program to monitor its effects. The program should also be implemented with other family members, such as children or grandchildren, to measure its effect on retirees.

In the future, we also recommend that the program use a blended approach, allowing it to be more accessible, and that people be more involved in the development of their skills for active and healthy ageing. It is recommended to implement the program using innovative methodologies and strategies, including blended sessions and informatics tools, the internet, and social media.

## 5. Conclusions

The REATIVA program was implemented with the EG_1_ and EG_2_ to determine its effectiveness. A control group (CG) consisting of recent retirees was also included but had no intervention. The participants of these groups measured the effectiveness of this program by completing the General Self-Efficacy Scale (GSE) and Positioning Scale for Adaptation to Retirement (EPFAR).

The REATIVA program proved to be efficient in accomplishing its purpose; namely, the promotion of the perception of general self-efficacy and positioning for adaptation to the transition to retirement. Its effectiveness was evident in the positive change in perceived self-efficacy identified in the retirees who participated in the program, suggesting that they now believe more in their competencies in starting, carrying out, and successfully performing specific tasks that may require effort and perseverance in the face of adversity.

The REATIVA program is being implemented in the healthcare surveillance of Portuguese new retirees with success. More studies are needed with larger groups. Further, the program is under review and adaptation in order to be implemented in other countries; namely, Portuguese-speaking (Brazil) and culturally similar (Spain) countries.

## Figures and Tables

**Table 1:** The efficiency of the REATIVA program General Self-Efficacy scale (Schwarzer and Jerusalem, 1995).

	Group		
	EG_1_ n = 15	EG_2_ n = 12	CG n = 28
Items	A2-A1 Median/SD	A2-A1 Median/SD	A2-A1 Median/SD
I can always manage to solve difficult problems if I try hard enough.	0.20/0.414	0.27/0.467	0.07/0.593
If someone opposes me‚ I can find the means and ways to get what I want.	0.14/0.352	0.36/0.674	0.10/0.378
It is easy for me to stick to my aims and accomplish my goals.	0.07/0.458	0.27/1.272	0.69/0.458
I am confident that I could deal efficiently with unexpected events.	0.46/0.640	0.45/0.934	−0.69/0.799
Thanks to my resourcefulness‚ I know how to handle unforeseen situations.	0.26/0.594	0.27/0.467	0.03/0.325
I can solve most problems if I invest the necessary effort.	0.14/0.352	0.55/1.036	0.03/0.731
I can remain calm when facing difficulties because I can rely on my coping abilities.	0.20/0.561	0.55/0.688	−0.03/0.325
When I am confronted with a problem‚ I can usually find several solutions.	0.14/0.352	0.27/0.647	−0.14/0.875
If I am in trouble‚ I can usually think of a solution.	0.07/0.458	0.36/0.505	0.00/0.620
I can usually handle whatever comes my way.	0.47/0.743	0.27/1.009	−0.20/0.860
TOTAL	0.19/0.219	0.36/0.488	−0.03/0.278
	*t* = 0.287/*p* = 0.005	*t* = 0.469/*p* = 0.033	*t* = −0.034/*p* = 0.510

**Table 2:** The efficiency of the REATIVA program Positioning Scale for Adaptation to Retirement (Loureiro et al., 2015).

		Group		
		EG_1_ n = 15	EG_2_ n = 12	CG n = 28
Dimensions	Items	A2-A1 M/SD	A2-A1 M/SD	A2-A1 M/SD
Retiree Status	I feel comfortable with my retiree status.	0.20/0.862	0.00/0.447	−0.07/0.842
	I can identify goals for my current life.	0.54/0.990	0.00/0.447	0.13/0.953
	I can identify goals for my future life.	0.66/1.113	0.09/0.302	0.07/0.998
	Being retired allows me to carry out projects.	0.53/1.125	0.36/0.674	0.07/0.842
Mental Health	I feel in harmony with my current life.	0.60/0.737	0.09/0.302	0.28/0.797
	I feel that I have more time for my activities.	0.26/0.594	−0.27/0467	0.17/0.805
	I use my time in a productive way.	0.27/0.594	−0.09/0.302	0.14/0.833
	I can manage everyday stress.	0.07/1.223	−0.27/0.467	−0.07/0.258
Support Network	I feel supported by my family.	−0.07/0.594	0.18/0.405	0.17/0.711
	The resources in my community are sufficient.	0.67/0.976	0.09/0.302	0.21/0.819
	I know how to have access to my community’s resources.	0.27/1.163	0.00/0.477	0.31/0.850
	I feel I have the necessary support when I need it.	0.27/0.884	0.18/0.405	0.00/0.655
Economic Management	I make a monthly plan of my expenses.	0.47/0.834	−0.09/0.302	0.34/0.857
	I know how to manage my money.	0.20/0.676	0.09/0.302	0.07/0.593
	I can set priorities for the purchases I make.	0.07/0.799	−0.18/0.405	0.03/0.823
	I know how to resist the pressure of consumer advertising.	0.27/0.594	−0.09/0.302	0.17/0.468
Health and Aging	I like myself the way I am.	0.34/0.816	0.00/0.447	0.14/0.516
	I feel fine.	0.06/0.961	−0.09/0.302	0.11/1.012
	I take care of myself.	0.20/0.941	0.09/0.302	−0.18/0.602
	I am afraid of growing old.	0.26/1.223	0.00/0.567	0.10/0.772
Family and Conjugality	I am satisfied with my marital relationship.	0.40/0.828	0.09/0.456	0.07/0.923
	When I decide with my partner, I achieve more and better.	0.73/0.961	0.09/0.302	0.10/0.900
	In my relationship, there is dialogue and sharing.	0.40/0.910	0.09/0.456	0.03/0.944
	I am sexually satisfied.	0.60/0.910	−0.09/0.302	0.03/0.906
Family and Parenting	I feel that I can count on my children.	0.33/0.976	−0.09/0.302	0.10/0.489
	I feel that I can continue to be useful to my children.	0.33/1.047	−0.18/0.603	0.00/0.535
	I feel that my grandchildren count on me.	0.60/0.828	0.09/0.456	−0.14/0.875
	My grandchildren make me happy.	0.73/0.884	0.00/0.447	0,21/0,726
Total		0.37/0.366	0.02/0.700	0.09/0.325
		*t* = 3.906/*p* = 0.002	*t* = 1.676/*p* = 0.125	*t* = 1.549/*p* = 0.132

## Data Availability

The data presented in this study are available on request from the corresponding author.
